# A City Shared Bike Dispatch Approach Based on Temporal Graph Convolutional Network and Genetic Algorithm

**DOI:** 10.3390/biomimetics9060368

**Published:** 2024-06-17

**Authors:** Ji Ma, Shenggen Zheng, Shangjing Lin, Yonghong Cheng

**Affiliations:** 1 School of Network Security, Jinling Institute of Technology, Nanjing 211169, China; 2 School of Economics and Management, Anhui Polytechnic University, Wuhu 241000, China; yonghongch1228@163.com; 3 Big Data Division, Beijing Logistics Technology Development Co., Ltd., Beijing 610101, China; shenggen_zheng@outlook.com; 4 School of Electronic Engineering, Beijing University of Posts and Telecommunications, Beijing 100876, China; linshangjing@bupt.edu.cn

**Keywords:** spatiotemporal demand forecasting, bike-sharing, T-GCN, scheduling optimization

## Abstract

Public transportation scheduling aims to optimize the allocation of resources, enhance efficiency, and increase passenger satisfaction, all of which are crucial for building a sustainable urban transportation system. As a complement to public transportation, bike-sharing systems provide users with a solution for the last mile of travel, compensating for the lack of flexibility in public transportation and helping to improve its utilization rate. Due to the characteristics of shared bikes, including peak usage periods in the morning and evening and significant demand fluctuations across different areas, optimizing shared bike dispatch can better meet user needs, reduce vehicle vacancy rates, and increase operating revenue. To address this issue, this article proposes a comprehensive decision-making approach for spatiotemporal demand prediction and bike dispatch optimization. For demand prediction, we design a T-GCN (Temporal Graph Convolutional Network)-based bike demand prediction model. In terms of dispatch optimization, we consider factors such as dispatch capacity, distance restrictions, and dispatch costs, and design an optimization solution based on genetic algorithms. Finally, we validate the approach using shared bike operating data and show that the T-GCN can effectively predict the short-term demand for shared bikes. Meanwhile, the optimization model based on genetic algorithms provides a complete dispatch solution, verifying the model’s effectiveness. The shared bike dispatch approach proposed in this paper combines demand prediction with resource scheduling. This scheme can also be extended to other transportation scheduling problems with uncertain demand, such as store replenishment delivery and intercity inventory dispatch.

## 1. Introduction

A bike-sharing system (BSS), as a typical form of shared economy in the field of intelligent transportation, provides a solution to the “last mile” problem in urban travel with its flexible and convenient usage characteristics [1]. However, due to the spatial and temporal fluctuations in user demand, the BSS network faces constantly changing station flow and bike quantities during operation, leading to supply–demand imbalances. In some areas, the number of bikes is relatively scarce, making it difficult to meet user demand, while in other areas, there is a severe surplus of bikes, even negatively impacting the traffic flow. Therefore, once bike-sharing is implemented in urban operations, its core challenge is the rebalancing problem, i.e., how to periodically redistribute bikes to better match the distribution of user demand and improve system efficiency. This involves enhancing user satisfaction, increasing bike turnover rates, and reducing bike idle rates. These issues are generally referred to as the “Bike-Sharing Rebalancing Problem” (BRP).

The BRP consists of two stages: bike demand forecasting and bike rebalancing. Bike demand forecasting involves predicting the flow of bikes based on the bike order data. The order data describe the start and end time and location (latitude and longitude) of each bike trip, reflecting the use of the shared bike. These data not only describe the number of bikes departing from and arriving at a specific area within a certain time period but also illustrate the flow relationship between different areas. The BRP can be divided into static BRP and dynamic BRP. Static BRP refers to the redistribution of bikes during a fixed time period (usually low-demand periods, such as nighttime), without considering real-time changes in user demand [2]. Dynamic BRP, on the other hand, involves redistributing bikes while considering real-time changes in user demand. This method requires the real-time monitoring of user demand and dynamically adjusting the redistribution plan according to the changes in demand. This paper analyzes the fluctuation in bike demand in Beijing, China, where bike demand is characterized by high density, high frequency, and high volatility. Real-time redistribution is crucial to avoid bike surpluses or shortages. Therefore, this work focuses on the dynamic redistribution scenario of bike-sharing rebalancing.

In the context of dynamic BRP, it is necessary to downsample the data for more effective demand forecasting. The selection of the time interval for downsampling is a challenge. Smaller time intervals facilitate immediate rebalancing but also lead to greater demand volatility, increasing the difficulty of forecasting. Additionally, smaller time intervals require considering the transportation distance limitations of redistribution vehicles during immediate redistribution to ensure they can complete the delivery within an effective redistribution time. The entire process requires real-time demand forecasting and redistribution optimization, posing high demands on the execution efficiency of algorithms.

To address these challenges, this study proposes several innovative methods. Firstly, we focus on interval-based demand forecasting. Bike demand is usually influenced by user mobility, showing temporal correlations similar to a Markov process. This study employs a Temporal Graph Convolutional Network (T-GCN) to predict the bike demand in both temporal and spatial dimensions and conducts comparative analysis across multiple time intervals. Secondly, when modeling the bike redistribution problem, we consider constraints such as vehicle capacity and travel time, providing a comprehensive problem model for interval-based bike redistribution. Finally, considering algorithm efficiency and applicability, we develop a cost-minimizing solution based on a genetic algorithm and validate its effectiveness through simulations. The main contributions of this paper are summarized as follows:

- We employ the T-GCN to predict bike demand by capturing spatiotemporal features and conducting comparative analysis across multiple time intervals. The analysis shows that the prediction algorithm performs well at a 30-min interval, which can be considered a reasonable redistribution interval in practical applications.
- When modeling the bike redistribution problem, we fully consider the complexity of real-time redistribution scenarios. The model takes into account different types of redistribution vehicles with different capacity constraints, the requirement for vehicles to complete the delivery process within a limited time, and the possibility of a station being visited by single or multiple vehicles simultaneously and repeatedly. These considerations make the model more realistic.
- We design a cost-minimizing solution based on a genetic algorithm and validate its efficiency and applicability through simulations. In the problem scenario, the genetic algorithm converges in approximately 50 generations, demonstrating high computational efficiency, and enabling rapid response or the handling of large-scale datasets. The algorithm shows good adaptability across different time redistribution scenarios within a day, effectively coping with dynamically changing demand and exhibiting high robustness.

The remainder of this paper is organized as follows: Section 2 reviews the related work in the areas of bike-sharing systems, demand forecasting, and redistribution optimization. Section 3 describes the problem and introduces the methods, including the T-GCN model for demand forecasting and the genetic algorithm for redistribution optimization. Section 4 presents the simulation experiments and result analysis using a dataset of bike orders from a bike-sharing operator in Beijing. Section 5 discusses the findings and future application directions of this study. Finally, Section 6 concludes the paper.

## 2. Related Work

### 2.1. Bike-Sharing Rebalancing Problem

The BRP is a special type of vehicle routing problem that includes both pickup and delivery tasks, allowing multiple visits to each station. In this context, a station can either be a pickup point or a delivery point but not both, making it a strongly NP-hard problem. The BRP can be categorized into station-based systems and free-floating systems, depending on whether docking stations are used. Additionally, it can be divided into static BRP and dynamic BRP based on whether the user activity is negligible [3]. Building on the single commodity picking traveling salesman problem (1-PDTSP), the capacitated vehicle routing problem (CVRP), and the multiple traveling salesman problem (M-TSP), Dell’Amico et al. [4] constructed a four-model mixed integer linear programming (MILP) model to formalize the BRP. To address these problems, they developed a customized branch-and-cut algorithm. In 2016, Kadri et al. [5] proposed a multi-station bike-sharing balancing system that took into account factors such as the station vehicle capacity, scheduling vehicle capacity, and time windows. Each station was to be visited once by a scheduled vehicle, and the goal was to minimize the total wait time in the unbalanced state of the station. In order to improve the search efficiency, a variety of upper-bound and lower-bound estimation methods were used and integrated into the branch-and-bound algorithm. Cruz et al. [6] discussed a specific BRP problem, where only one vehicle was available, and the goal was to find the lowest cost route that met the needs of all stations while not violating the no-load running and maximum vehicle capacity load limits. To solve this problem, a heuristic algorithm based on iterative local search was proposed. Pal et al. [7] proposed a mixed integer linear programming (MILP) model to deal with multiple vehicles, allowing vehicles to access the same node multiple times for new static BRP problems, and introduced mixed nested large neighborhood search and variable neighborhood descent algorithms for solving. Schuijbroek et al. [8] presented a MILP clustering problem that decomposed the multi-bike rebalancing problem into a single-bike problem and combined service-level requirements with vehicle routing optimization. The nonstationary queuing system model was used to determine the inventory demand of each station, and the heuristic algorithm of "clustering before routing" was used to solve the problem. The Maximum Spanning Star approximation method was used to estimate the path cost, which improved the calculation efficiency and understanding accuracy by eliminating cuts. The results showed that the proposed heuristic algorithm was superior to the traditional mixed integer programming and constraint programming methods in terms of computational efficiency and quality. Guo et al. [9] analyzed the spatiotemporal flow characteristics of bicycles in the BRP, used hierarchical clustering to determine the similarity of travel between stations, and formed groups of stations. The research was divided into two aspects: intra-group rebalancing and inter-group rebalancing. Finally, a genetic algorithm was used to minimize the total driving distance of the rebalanced vehicles to achieve a cost-effective static rebalancing strategy. Vincent et al. [10] applied the traditional vehicle routing problem to crowdsourced drivers (ODs) to complete distribution tasks. Each customer needs to be served, the vehicle needs to balance flow and capacity limits, and the crowdsourced driver can only serve one customer. A simulated annealing (SA) algorithm was used to solve the problem, and the experimental results showed that it was better than the cplex exact solver.

The research on the BRP has mainly focused on the static balancing problem: in static scheduling, the demand for bicycles is fixed, the scheduling operation is performed at night, and the number of bicycles is not considered until the end of rebalancing. The dynamic adjustment and balance problem needs to consider the rapid changes in the state of the shared bicycle system, which is suitable for the daytime when the demand for bicycles is more frequent. The scheduling problem of dynamic shared bicycles mainly includes two aspects: demand forecasting and scheduling optimization [11]. In the demand forecasting phase, rapid forecasting of bicycle demand at specific times and regions is combined with traffic flow forecasting by analyzing historical user order data. Then, according to the generated demand prediction, the dispatching optimization is carried out; that is, the bicycle scheduling task is strategically arranged to minimize the cost and complete the bike-sharing rebalancing.

### 2.2. Traffic Flow Forecasting

In the field of traffic flow forecasting, early solutions were predominantly recognized for their reliance on the historical average method. These methods include the Autoregressive Integrated Moving Average (ARIMA) [12], the Seasonal Autoregressive Integrated Moving Average (SARIMA) [13], and the Multivariate Time-Series Models [14], among others. These approaches gained widespread application in traffic flow forecasting due to their simplicity and relatively high predictive accuracy. However, these algorithms exhibit significant limitations when faced with complex, nonlinear, and high-dimensional traffic flow data. To address the limitations, several advanced time series forecasting algorithms have been developed. They leverage modern computational techniques to capture complex patterns and improve prediction accuracy. Among these, machine learning algorithms, such as Support Vector Regression (SVR) [15] and Random Forests, have been increasingly applied due to their ability to model nonlinear relationships and learn from large datasets. Furthermore, deep learning techniques, including Long Short-Term Memory (LSTM) networks [16], Gated Recurrent Unit (GRU) networks [17], and Temporal Convolutional Networks (TCNs) [18], have shown significant promise in handling the complexities of time series data by capturing long-term dependencies and nonlinear patterns more effectively than traditional methods. Building on these advancements, recent innovations in deep neural networks have introduced even more sophisticated models for traffic flow prediction. Qu et al. [19] proposed an end-to-end hybrid deep learning network adapted to traffic flow data. It featured an online self-learning mechanism to address data imbalances and mitigate overfitting. Meng et al. [20] introduced an attention mechanism to address the issue of the varying influence of input characteristics at different times on the prediction of traffic flow, showing that this enhancement significantly improved the prediction accuracy compared to standard time series methods. Sadeghi-Niaraki et al. [21] introduced a short-term traffic flow prediction model, an enhanced Elman recurrent neural network (GA-MENN). The model used genetic algorithms (GA) to refine ELM’s hyperparameters and integrate various features, such as weather conditions, working days, and specific times, to improve prediction accuracy.

However, these traditional models often overlook the spatial dependence of the traffic flow, leading to the integration of Convolutional Neural Networks (CNNs) to extract the spatial characteristics of the road network. As a result, hybrid models that combine different forecasting techniques, such as CNN-LSTM hybrid models, have been particularly effective in traffic flow forecasting, leveraging the strengths of each approach to capture a wider range of patterns in traffic data. Zhang et al. [22] introduced a model for short-term traffic flow prediction, which was based on convolutional neural networks (CNNs), a subset of deep learning technology. This model employed a unique algorithm called the spatiotemporal feature selection algorithm (STFSA), which decided how to incorporate historical data, including both temporal and spatial information, as inputs. After the model’s construction, its accuracy was tested by comparing the predicted traffic flows with real-world data. Du et al. [23] proposed a simplified deep-learning method for traffic prediction. It used one-dimensional Convolutional Neural Networks (1D CNNs) and Gated Recurrent Units (GRUs) with attention mechanisms. The 1D CNNs captured local trends, while the GRUs identified long-term patterns. The method was enhanced by a multimodal deep learning framework, combining multiple CNN-GRU-Attention modules to merge different traffic data, improving the prediction accuracy. Graph Neural Networks (GNNs) have recently gained prominence, particularly for capturing the “non-Euclidean structure” of traffic networks. Attention mechanisms and convolutional strategies have been incorporated into GNNs, resulting in Graph Attention Networks (GANs) and Graph Convolutional Networks (GCNs). Zhao et al. [24] proposed the Temporal Graph Convolutional Network (T-GCN), merging GCN’s spatial feature extraction with GRU’s time series prediction to simultaneously capture spatial and temporal dependencies in traffic data. Bai et al. [25] introduced an attention mechanism into the T-GCN, creating the Attention-Based Temporal Graph Convolutional Network (A3 T-GCN), which adjusted the weights of different time points and integrated global time information to enhance the prediction accuracy. Zhu et al. [26] proposed the Attribute-Enhanced Temporal Graph Convolutional Network (AST-GCN) for traffic flow prediction, incorporating external factors like weather and Point of Interest (POI) distribution, demonstrating improved prediction accuracy and model explainability.

### 2.3. Dispatching Optimization

The dispatching optimization phase of shared bikes can be regarded as the Pickup and Delivery Vehicle Routing Problem (PDVRP). The essence of this problem is a combinatorial optimization problem, and it is difficult to solve and obtain an optimal solution within a reasonable time. With the continuous development of computing power, using a computer heuristic algorithm has become an effective way to solve the problem. Heuristic algorithms such as a genetic algorithm, an ant colony algorithm, and an artificial neural network have shown high application potential. Liu et al. [27] established the income function of bike delivery at the drop-off point with reference to the newspaper delivery model. Based on this, this work established the replacement path planning model of shared bikes with pickup and delivery and used a genetic algorithm to solve it. Xv et al. [28] and others combined chaos theory and an ant colony system to improve the ant colony algorithm to solve the process planning problem of the static dispatching of shared bike services. Wang et al. [29] developed a new model for BRP using the NSGA-II algorithm. The model analyzed user travel patterns through order data, segmented operating areas into interconnected communities, and evaluated submarkets within these communities. It then optimized the number of transport vehicles and dispatch points to minimize the costs and maximize bike utilization. Cui et al. [30] proposed a scheduling model to address the cost and service loss problems in the operation of shared bikes. The model took truck activation, route selection, and vehicle scheduling as the core variables. To transform the model into a linear programming problem, a linearization method was devised. Additionally, a greedy strategy based on an artificial swarm algorithm was developed to efficiently solve large-scale scheduling problems. Through numerical experiments, a detailed analysis of the problem and the algorithm performance was conducted, providing a scientific basis for shared bike scheduling decisions. Xu et al. [31] suggested a bike-sharing dispatch strategy that encouraged users to ride idle shared bikes (red envelope bikes) to high-demand areas for scheduling. Users received a red packet reward for completing the task. The study devised a hybrid integer programming model and a hybrid tabu search algorithm to efficiently solve the large-scale scheduling problem.

## 3. Problem Description and Methodology

### 3.1. Problem Description

After a period of natural operation, when the distribution of bikes in the bike-sharing system no longer aligns with user needs, it becomes necessary to activate the dispatching mechanism. The dispatching problem can be divided into two stages: demand forecasting and dispatching optimization. In the demand forecasting stage, future bike demand for specific times and regions is predicted through analysis of historical user order data. The demand forecasting model can be viewed as vertical time series forecasting; however, due to the mobile nature of shared bikes, their spatial locations play a crucial role. Therefore, it is essential to consider both spatial and temporal dependencies in this stage. In the dispatching optimization stage, optimal operational efficiency can be achieved by strategically arranging the bike distribution and dispatching based on the predicted demand. The objective of this process is to enhance resource utilization efficiency while maintaining service levels to better meet users’ travel needs.

### 3.2. Demand Forecasting Model

**Figure 1:**
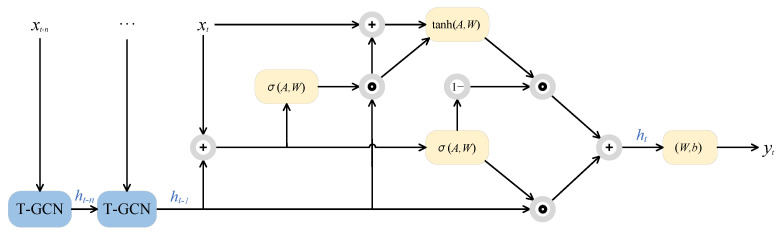
The architecture of the T-GCN model.

The T-GCN model is a neural network-based approach for traffic prediction, designed to capture both spatial and temporal features of the data simultaneously. As illustrated in Figure 1, the T-GCN employs a variant of the recurrent neural network architecture known as Gated Recurrent Unit (GRU), integrating graph convolution methods from Graph Convolutional Networks (GCNs) into the computation of the update gate, reset gate, and hidden layer output [24].

GCNs represent a developmental branch of a CNN tailored for modeling graph structures. By applying convolutional operations on the nodes of a graph, GCNs aim to effectively extract and propagate node features [32]. Through inter-node interactions and information dissemination, GCNs are capable of learning intricate patterns and relationships within graphs, facilitating efficient feature extraction and classification of graph data. GRUs constitute an enhanced variant of Recurrent Neural Networks (RNNs), devised to address the issues of vanishing and exploding gradients in long-term memory and backpropagation. Compared to LSTM networks, GRUs streamline their architecture, employing solely update and reset gates to govern units, thereby reducing the number of network parameters [33]. This simplified design renders GRUs more efficient in handling sequential data, while adeptly capturing temporal features. T-GCNs integrate GCN convolution within GRU computations, leveraging GCN’s convolutional traffic topology to capture spatial dependencies and GRU’s learning of historical traffic data variations to capture temporal dependencies. The data contain spatiotemporal relationships of traffic flow, and the use of T-GCN can more fully exploit the information contained in the data.

In the context of the T-GCN model, we define an unweighted directed graph G=(V,E) to represent the topological structure of traffic flow between nodes in a traffic network, where *V* is the set of nodes, each representing a grid in the network, and *E* is the set of edges, with edge weights denoting the connectivity between grid nodes in terms of traffic flow. We utilize the adjacency matrix *A* to denote the connection weights between nodes, $A\in R^{N\times N}$, where *N* is the number of grid nodes. We construct a feature matrix x $X^{P\times N}$ where each node’s business demand at *p* time steps is represented. We consider the traffic information on the road network as node attribute features, denoted as *X*. At any time step *i*, the business demands of all nodes form an attribute vector $\vec{x},i=1,2,\ldots ,P$.

The goal of the T-GCN is to learn a mapping function *f* and calculate the predicted value of the next *m* time steps according to graph *G* and the feature matrix *X*, as shown in the formula:

(1) $$\left[x_{t+1},x_{t+2},\ldots ,x_{t+m}\right]=f\left(G;\left(x_{t-n},\ldots ,x_{t-1},x_{t}\right)\right).$$

The T-GCN draws on the graph convolution operation of a GCN when learning spatial features, and the formula of a GCN can be expressed as

(2) $$f\left(X,A\right)=\sigma (\hat{A}Relu\left(\hat{A}XW_{0}\right)W_{1})$$

(3) $$\hat{A}={\tilde{D}}^{-1/2}\tilde{A}{\tilde{D}}^{-1/2}$$

(4) $$\tilde{A}=A+I_{n}$$

where *W* is the weight matrix, σ and Relu are the activation functions, $\hat{A}$ is the Laplacian matrix, $\tilde{D}$ is the degree matrix, and $I_{n}$ is the identity matrix.

When learning temporal features, the T-GCN utilizes the GRU model, where $g\left(A,X\right)=\hat{A}X$ is defined. Graph convolution operations are performed when computing the update gate, reset gate, and hidden layer output of the GRU. The specific formulas are as follows:

(5) $$r_{t}=\sigma \left(W_{r}\left[g\left(A,X_{t}\right),h_{t-1}\right]+b_{r}\right)$$

(6) $$u_{t}=\sigma \left(W_{u}\left[g\left(A,X_{t}\right),h_{t-1}\right]+b_{u}\right)$$

(7) $${h_{t}}^{\prime }=\tanh\left(W_{c}\left[g\left(A,X_{t}\right),\left(r_{t}*h_{t-1}\right)\right]+b_{c}\right)$$

(8) $$h_{t}=u_{t}*h_{t-1}+\left(1-u_{t}\right)*{h_{t}}^{\prime }$$

where *r* is the update gate, *u* is the reset gate, ${h_{t}}^{\prime }$ is the updated value, and $h_{t}$ is the final hidden layer output, and together with the features of the next moment, it is the input of the next moment.

### 3.3. Dispatching Optimization Model

#### 3.3.1. Notation Descriptions

To facilitate the description of the dispatching optimization model of shared bikes, we defined the notations and variables, as shown in Table 1.

#### 3.3.2. Problem Definition and Hypothesis

Suppose a city is divided into *N* nodes, i∈1,2,3,…N, where each node represents a region. $Out_{i}$ indicates the number of shared bikes on node *i* at the start of dispatching. $In_{i}$ indicates the number of shared bikes entered on node *i* at the end of dispatching. Therefore, the bike demand of each node during the dispatching period is

(9) $$G_{i}=Out_{i}-In_{i}.$$

When $G_{i}$ is equal to 0, the node is in a self-balancing state, and there is no dispatching demand. When $G_{i}<0$, the number of bikes in the node exceeds the demand overflow, and the node needs to be transferred out of the vehicle. When $G_{i}>0$, the bike node does not meet the demand, and the bike needs to be transferred to maintain balance.

The BRP can be described as follows: in a given dispatching area, there are multiple dispatching vehicles, and the loading capacity of these vehicles is limited. The task of dispatching is to start these vehicles from the specified starting point, according to certain rules, and complete a series of bike recycling and delivery operations in the dispatching period, until the completion of the entire dispatching work. Specifically, the dispatching vehicle needs to reach the node with $G_{i}>0$ to pick up the excess bikes and the node with $G_{i}<0$ to deliver the bikes. The dispatching vehicle should complete the work within a certain time interval. Therefore, the maximum driving distance constraint is set for the dispatching vehicle to be able to complete the dispatching work within the time interval as far as possible.

In addressing the above issues, we propose the following assumptions:

1. Dispatching is carried out in the form of task reward release, dispatching vehicles have no fixed cost, and only dispatching vehicles within the starting point of the path node can accept tasks.
2. The distance between nodes is measured by the Manhattan distance.
3. There are very few orders during the dispatching period; so, the bike movement has little impact on the overall system.
4. The starting point of the dispatching car must be a node with $G_{i}>0$, and the endpoint must be a node with $G_{i}<0$.
5. In a dispatch trip, vehicles can pick up and drop bikes at multiple nodes, but all bikes must be completely dropped by the end of the dispatch trip.
6. In each pick-up area, the number of bikes picked up by the vehicle should meet the pick-up requirements as much as possible without exceeding the vehicle’s load capacity.
7. At each delivery area, the vehicle needs to unload the required bikes for the area or all the bikes, but the unloaded bikes cannot exceed the amount required by the area.

#### 3.3.3. Mathematical Model

Considering that there are multiple dispatching vehicles that need to dispatch cooperatively and that the load and maximum distance limits need to be considered, the dispatching problem can be regarded as a VRP problem with a repeatable path with capacity and distance constraints. The goal of the dispatching model is to ensure the lowest dispatching cost for operators. The mathematical model is as follows:

(10) $$\begin{array}{cc} \; & \min Z=\sum_{m=1}^{M}\sum_{i=1}^{N}\sum_{j=1}^{N}C\cdot x_{mij}\cdot d_{ij}\;\\ \; & such\;that\;\left\{\begin{array}{c} \sum_{i=1}^{N}\sum_{j=1}^{N}x_{mij}d_{ij}\leq D,\;\;for\;i,j=1,2,\ldots ,N\\ \sum_{i=m}^{M}\sum_{i=1}^{N}r_{mi}=\sum_{i=m}^{M}\sum_{i=1}^{N}p_{mi}\\ \sum_{m=1}^{M}\left(r_{mi}+p_{mi}\right)=G_{i}\\ r_{mi},p_{mi}\leq V_{m}\\ d_{ij}=|x_{i}-x_{j}\left|+\right|y_{i}-y_{j}|\\ x_{mij}\in \left\{0,1\right\} \end{array}\right. \end{array}$$

where the objective function aims to minimize the total dispatching cost, which is solely contingent upon the number of dispatched vehicles and the transportation distance, given that dispatching schemes are disseminated on a task-by-task basis, devoid of fixed dispatching costs. The first constraint restricts the maximum dispatch transportation distance of vehicles to not exceed the maximum transportation distance. The second constraint denotes that all recalled vehicles must be deployed entirely. The third constraint stipulates that the number of vehicles recalled or deployed cannot surpass the demand volume of the nodes. The fourth constraint restricts the dispatched vehicles for retrieval or deployment to not exceed their individual maximum capacity limit. The fifth constraint specifies that the distance between nodes adheres to the Manhattan distance. Lastly, the final constraint mandates that $x_{ij}$ is a binary variable.

#### 3.3.4. Problem Solving

The idea of the dynamic path planning optimization model is applied to BRP, which is a typical NP-hard problem and difficult to solve by an optimization algorithm. In the field of path planning, the genetic algorithm has attracted much attention because of its powerful optimization ability. The basic idea is to imitate the law of natural selection, to express the initial solution through a mathematical chromosome, and to achieve optimization through an evolutionary operation. The genetic algorithm has excellent capability for parallel computation and a global search. Aiming at the dispatching optimization problem, we designed the genetic algorithm as follows:

1. Encode
  In this work, we have chosen the encoding of natural numbers to be consistent with the encoding of nodes, such as R∈(1,2,3,…,N), which indicates a simple dispatching path. The random full permutation generating path is used as the initial population.
2. Decode
  On the dispatching path, the dispatch vehicle may exhibit one of four behaviors when passing through node *i*, which are as follows:
  (a) The vehicle will pick up all extra bikes in node *i*.
  (b) The vehicle will pick up a portion of bikes at pick-up node *i*, potentially reaching its full load capacity during the dispatch process.
  (c) The dispatch vehicle drops all its bikes at node *i*, yet the demand at node *i* remains unmet.
  (d) The vehicle unloads enough bikes to meet the demand at node *i*, but there are still excess bikes remaining on the dispatch vehicle.
  To deal with these behaviors, the following decoding rules are formulated:
  (a) Alternate dispatching: Based on bike demands, nodes are categorized into pick-up nodes and delivery nodes, representing positive and negative demands, respectively. Dispatching operations involve collecting bikes from pick-up areas to reduce excess and delivering them to delivery areas to meet demands. In each operation, the dispatch vehicle must be fully loaded first and completely unloaded by the end of the dispatch.
  (b) Chromosome expansion: Nodes that meet the requirements are removed from the partition list. If behaviors (b) and (c) are encountered, indicating that the requirements for node *i* are not met, the requirements for node *i* are retained and updated in the partition. The scheduler continues to dispatch based on the partition and expands the chromosome to indicate that the path can be repeated. For example, the chromosome R(1,2,3,…,N) is extended to R(1,2,3,2,4,…,N), indicating that the dispatch vehicle did not meet the requirements of node 2 on the first visit and returns to node 2 after processing node 3.
  (c) Chromosome splitting: When the dispatch vehicle completes one cycle of alternate dispatching and the next cycle exceeds the maximum distance limit, the chromosome is split. For example, splitting the chromosome (1,2,3,2,4,…,N) results in (1,2,3,0,2,4,…,8,0,9,10,…,N), indicating that the dispatch path for the first vehicle is (1−2−3), the second vehicle’s path is (2−4−…−8), and the third vehicle’s path is (9−10−…−N).
3. Fitness Function
  The fitness function is defined as the total dispatching cost of shared bikes, aligning with the objective function. A smaller fitness value indicates a better solution.

The pseudo-code of the process of genetic algorithm solving dispatching problem is shown in Algorithm 1:
**Algorithm 1** Genetic Algorithm**Input:** GAP: a matrix represents the number of nodes required, *P*: population size, pc: crossover probability, pm: mutation probability, *T*: iterations**Output**: Routes: dispatch routes. Cost: dispatching cost**begin**  1: Initialize population P(0) with randomly generated chromosomes using natural number encoding;  2: t←0;  3: **while** (t≤T) **do**  4:       **for** i←1
**to**
*P* **do**  5:          Evaluate fitness of each chromosome in P(t);  6:       **end for**  7:       **for** i←1
**to**
*M* **do**  8:         Select parent chromosomes for crossover from P(t);  9:       **end for**10:       **for** i←1
**to**
M/2 **do**11:          Perform crossover operation on selected parent chromosomes;12:       **end for**13:       **for** i←1
**to**
*M* **do**14:          Perform mutation operation on chromosomes in P(t);15:       **end for**16:       **for** i←1
**to**
*M* **do**17:          Decode chromosomes in P(t) using the decoding rules;18:          Update chromosomes in P(t) according to the decoded results;19:       **end for**20:       t←t+1;21: **end while**22: **end**

## 4. Experimental Analysis

### 4.1. Experimental Data

#### 4.1.1. Data Sources

In this study, the order data of shared bikes in Beijing were adopted. The data set covers the use records of 376,556 bikes by 279,811 million users within 14 days, totaling more than 3 million records. Each data record contains information such as the order start time, start position, and end position, as shown in Table 2.

#### 4.1.2. Data Preprocessing

The start and end locations of the orders in the raw data were encrypted, and we first decrypted them using the geohash algorithm. We then organized the data in detail based on 30-min intervals. For missing data, we used the populating strategy of the historical average at that moment, while removing the absence of data throughout the day.

**Figure 2:**
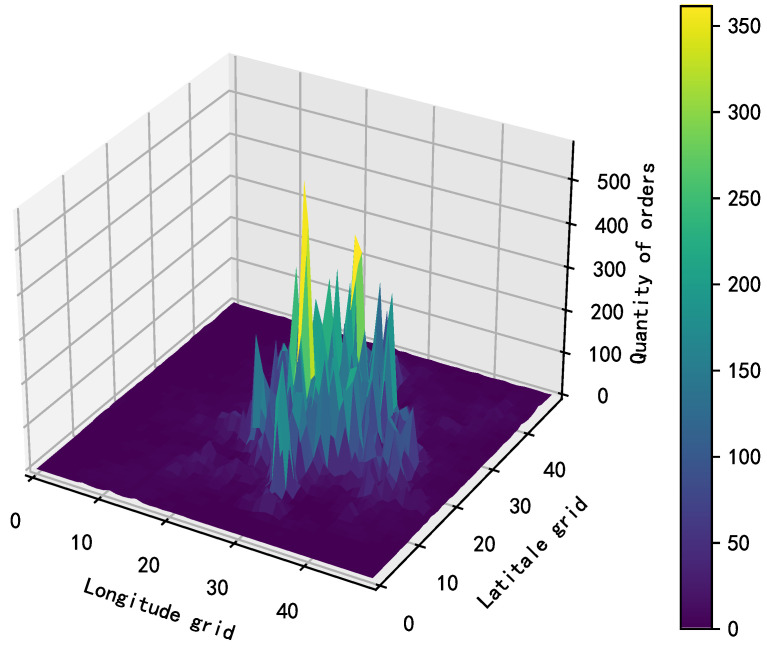
Heat map of bike demand.

We carefully divided the bike flow area into 50 × 50 grids to better understand and analyze the distribution of bike orders. Different bike orders were assigned to the corresponding grid using the order’s starting geographical location coordinates. Based on this, we drew Figure 2 according to the historical order quantity of each grid, where the x and y axes represent the grid’s horizontal and vertical numbers, respectively, and the z-axis represents the number of orders. According to the results of the chart, the order data were mainly concentrated in the central zone, indicating that there was more frequent bike use in this area. This phenomenon reflects the physical location corresponding to urban centers, which have higher population density and commercial activity levels, resulting in an increased demand for bikes. In addition, central areas generally have more transportation hubs and important destinations, which may stimulate people to choose cycling as a means of travel more frequently.

**Figure 3:**
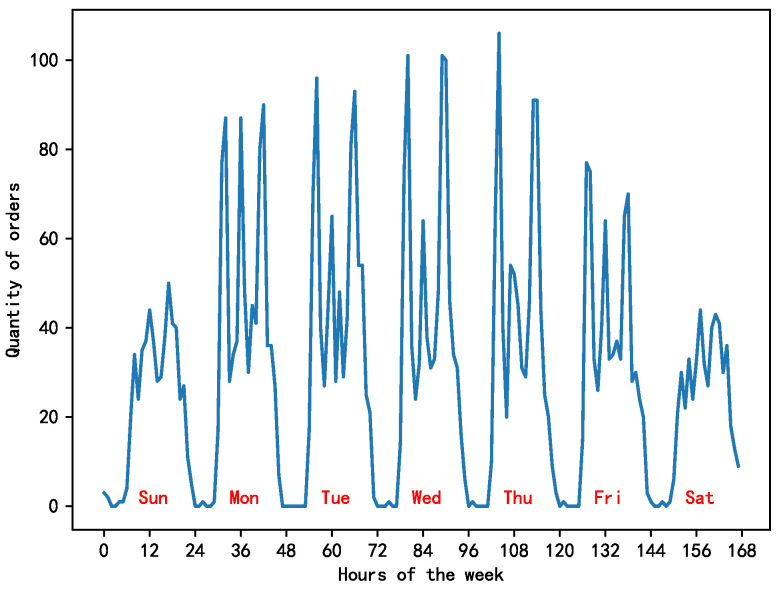
Cycle demand changes over the week/weekday.

In this context, a grid was selected at random to depict the bike demand pattern over a one-week period, as shown in Figure 3. The left side of the figure displays the variations in bike demand throughout the week, whereas the right side represents the changes in the average daily demand for bikes during the working week. The horizontal axis shows the time, and the vertical axis shows the number of bikes ordered. The observations indicate that the number of bike orders on weekdays exhibited three distinct peaks: 7–9 AM, 11 AM–1 PM, and 5–7 PM. These times correspond to the morning rush hour, lunch break, and evening rush hour in Beijing, respectively. In contrast, weekend demand patterns did not show a notable surge, likely due to the more varied and less structured nature of weekend travel times.

To train and evaluate the requirements prediction model, we divided the data set according to typical practice, using 80% of the data as the training set and the remaining 20% as the testing set. Our task was to predict the change in bike flow at the next 30th, 60th, and 120th minute nodes.

### 4.2. Performance Indicators

In assessing the performance of the demand forecasting model, three evaluation metrics were employed: Root Mean Square Error (RMSE), Mean Absolute Error (MAE), and Coefficient of Determination ($R^{2}$). These metrics quantify the difference between the actual bike demand samples (*y*) and the forecasted demand values ($\hat{y}$). Specifically, the RMSE and MAE measure the magnitude of the prediction error, with smaller values indicating better predictive accuracy. Meanwhile, $R^{2}$ was calculated to determine how well the forecasted outcomes represented the empirical data. An $R^{2}$ value close to 1 indicates a strong alignment between the predicted and actual values. The detailed evaluation indicators are as follows:

(11) $$RMSE\left(y,\hat{y}\right)=\sqrt{\frac{1}{n}\sum_{i=1}^{n}{\left(y_{i}-{\hat{y}}_{i}\right)}^{2}}$$

(12) $$MAE\left(y,\hat{y}\right)=\frac{1}{n}\sum_{i=1}^{n}\left(\left|y_{i}-{\hat{y}}_{i}\right|\right)$$

(13) $$R^{2}\left(y,\hat{y}\right)=1-\frac{\sum_{i=1}^{n}{\left(y_{i}-{\hat{y}}_{i}\right)}^{2}}{\sum_{i=1}^{n}{\left(y_{i}-{\bar{y}}_{i}\right)}^{2}}$$

### 4.3. Demand Forecasting Experiment

In this experiment, we set the learning rate of the T-GCN to 0.001, with a batch size of 32 and a time step of 4. Three models were constructed, each aimed at single-step, two-step, and four-step forecasting, corresponding to predicting bike demand for 30, 60, and 120 min ahead, respectively. The training iterations were set to 50. Within the T-GCN framework, the hidden size and time step of the GRU significantly influence the model’s predictive performance. We selected the optimal hidden sizes from the candidate set [8, 16, 32, 64, 128, 256]. As depicted in Table 3, our results demonstrated that the optimal hidden layer sizes were 64, 64, and 128, respectively, corresponding to the best predictive performance.

### 4.4. Dispatching Optimization Experiment

To refine the fundamental parameter configuration within the framework of the BRP model, this study employed three distinct vehicles characterized by capacities of 20, 15, and 10, respectively, with the aim of streamlining the complexity inherent in the problem. The dispatching framework is structured around task release dynamics; hence, there is no fixed dispatching cost. The unit distance cost and maximum distance traveled are consistent for all types of dispatch vehicles. See Table 4 for the specific parameter values.

In this experiment, the genetic algorithm was designed to solve the BRP, and the specific selection, crossover, and mutation operations were as follows:

In the selection phase, we applied the roulette method and introduced an elite retention strategy. First, the individuals in the population were ranked according to their fitness, and the top individuals were promoted directly to the next generation. The remaining individuals were screened using the roulette selection method, which ensures search efficiency while maintaining population diversity, helping the algorithm to strike a balance between a global and local search.

In the crossover phase, we used the partially mapped crossover operator (PMX) strategy. First, two parent individuals were randomly selected as the basis for the crossing. Then, two different intersections were identified, and the gene segments to be exchanged were defined. Subsequently, the gene fragments within the intersection of the two parent chromosomes were exchanged to form two primary parent chromosomes. After the exchange, there may be a problem with repeated visits to the city. To solve this problem, we checked whether there were duplicate cities in each offspring and corrected them according to a specific method: for each duplicate city, its original location was found in another offspring, and the genetic sequence between the position of the duplicate city in the current offspring and the position it should be in was replaced to maintain chromosome continuity and legitimacy. If a gene duplicate that caused the target location was replaced, the process was performed recursively until all the duplicates were eliminated. After processing, both children were legitimate TSP solutions, and there was no problem of city duplicate access.

In the mutation phase, we implemented the exchange mutation strategy. We randomly selected two different locations on the chromosome and swapped the cities at these two locations to form a new chromosome.

The genetic algorithm parameters included the population size, crossover probability, and mutation probability. The specific value settings for these parameters are shown in Table 5.

### 4.5. Results

#### 4.5.1. Experimental Results of the Demand Prediction

The T-GCN model was employed to forecast bike demand across various time intervals: 30 min, 60 min, and 120 min. Its effectiveness was rigorously assessed using key metrics, including the Mean Absolute Error (MAE), the Root Mean Square Error (RMSE), and the Coefficient of Determination ($R^{2}$). The experimental results are shown in Table 6. For the 30-min prediction window, the T-GCN model showcased outstanding performance, yielding a low MAE of 0.4786, a minimal RMSE of 2.2175, and a high R2 value of 0.8974. These metrics underscored its remarkable accuracy and fitting capability in short-term time series forecasting tasks. However, as the prediction horizon extended to 60 min and subsequently to 120 min, the model’s performance witnessed a noticeable decline. In the 60-min forecast, the MAE increased to 5.342, the RMSE increased to 2.4909, and the R2 slightly decreased to 0.8741. Furthermore, in the 120-min forecast scenario, the MAE further rose to 0.8147, the RMSE rose to 3.7291, and the R2 dropped to 0.7131. This trend suggests that the T-GCN model may encounter greater challenges in longer-term predictions, resulting in a reduction in its predictive accuracy. However, it is worth noting that the model demonstrated excellent performance in short-term forecasting, indicating its proficiency in capturing immediate trends and patterns.

**Figure 4:**
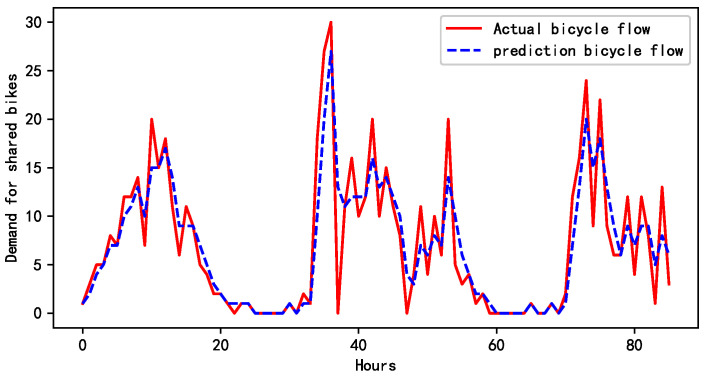
The T-GCN predicts changes in the demand data of bikes within nodes.

Figure 4 illustrates a comparison between the demand forecast for shared bikes based on the 60-min interval data and the actual sample data for a specific area. A detailed analysis of the chart reveals that the T-GCN model effectively captured the cyclical variations in the demand for shared bikes over this time frame. The model’s prediction outcomes exhibited a high level of agreement with the real-world sample data. This highlights the T-GCN model’s strong performance in time-series forecasting tasks, particularly its ability to identify the cyclical patterns of demand within a short period. These findings reinforce the efficacy of the T-GCN model in predicting the demand for shared bikes, providing a quantitative foundation for further research into the optimization of BRP.

#### 4.5.2. Experimental Results of the Dispatching Optimization

**Figure 5:**
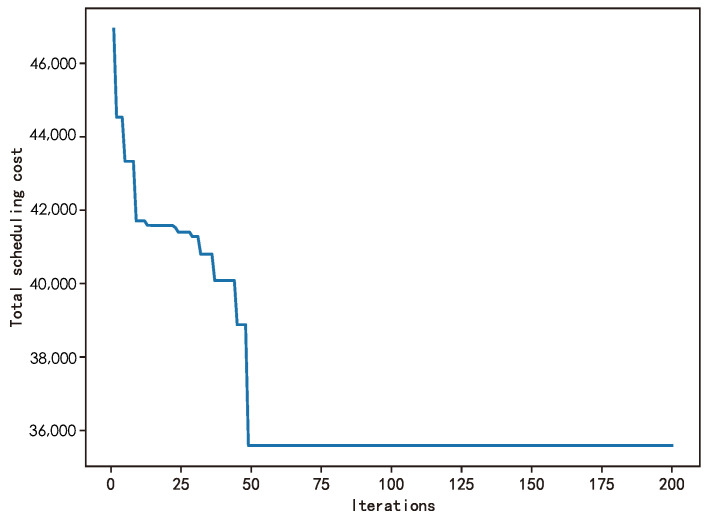
The genetic algorithm’s iteration results in the dispatching model.

In the scheduling optimization experiment, the iterative effect of the genetic algorithm is shown in Figure 5. In the figure, the horizontal axis represents the number of iterations, and the vertical axis represents the scheduling cost, that is, the fitness function value. It can be observed from the figure that with the increase in the iterations, the genetic algorithm gradually optimized the scheduling scheme and gradually approached the optimal solution. When the number of iterations reached 50, the scheduling optimization model showed an obvious convergence trend, which indicated that the algorithm was close to or reached the optimal solution.

To evaluate the robustness of our genetic algorithm across various scheduling scenarios, we conducted a comprehensive analysis from 7 AM to 10 PM. We recorded the convergence iterations of the algorithm for half-hour and one-hour scheduling intervals, as detailed in Table 7. The table shows that the genetic algorithm exhibits similar convergence behavior for both half-hour and one-hour scheduling intervals. Specifically, the algorithm typically achieves convergence within relatively few iterations for both half-hour and one-hour scheduling problems. These findings reflect the algorithm’s efficiency and responsiveness in addressing complex scheduling problems involving 2500 nodes. Furthermore, the algorithm demonstrates stable convergence across peak and off-peak hours. This performance underscores its adaptability to dynamic scheduling scenarios, substantiating its reliability and effectiveness in practical applications.

**Figure 6:**
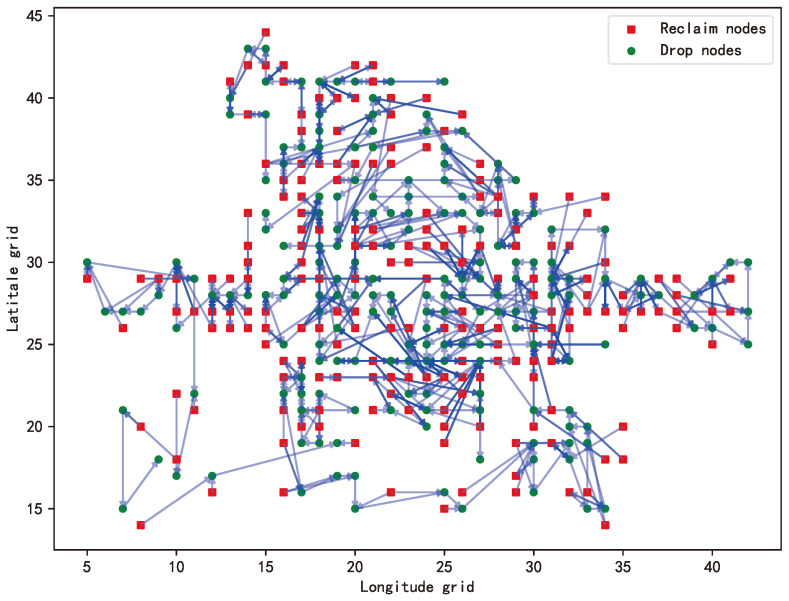
Dispatch vehicle transport routes. The directed edges represent the bicycle dispatch routes.

The final experimental results showed that the total cost required to achieve the optimal solution was CNY 35,588, and the number of dispatch vehicles involved was 531. The optimization scheme showed remarkable benefits in practical application. In the final dispatching path shown in Figure 6, there is a two-dimensional coordinate system, wherein the longitudinal grid sequence is represented along the horizontal axis, denoting east–west grid progression, while the latitudinal grid sequence is portrayed along the vertical axis, signifying north–south grid progression. The optimization results of the vehicle dispatching are presented in a color-coded manner, with red square markers indicating nodes with excess vehicles and green circular markers indicating nodes with insufficient vehicles. The figure describes in detail that the dispatching vehicle starts from the red square node and ends with the green circular node, alternating between the red square node and the green circular node. This means that the dispatching vehicle can effectively carry out the “recycling–dropping” operation. It is worth noting that the dispatching vehicle can visit the same node several times, and the same node can also be visited by multiple dispatching vehicles, which makes the dispatching task closer to the actual dispatching mode. This strategy not only shows the high flexibility of the dispatching vehicle in the execution of the task but also reflects its optimization performance in path planning. This dispatching strategy can maximize the use efficiency of vehicle resources and complete dispatching tasks in a low-cost and efficient way.

## 5. Discussion

This study focused on the dynamic bike-sharing rebalancing problem, designing a demand forecasting and bike dispatch optimization mechanism. It employed T-GCN for demand forecasting and used genetic algorithms for bike dispatch. The study divided the urban area into 2500 grids based on latitude and longitude as nodes of the graph and established behavioral links between grids as edges of the graph through the starting positions in orders, thus generating graph data with 2500 nodes. The T-GCN utilized GCN layers to extract spatial features from the graph and embedded them into the GRU to extract temporal features, thereby achieving spatiotemporal scale bike demand forecasting. During the bike dispatch process, the model assumed a sufficient number of carrier vehicles for bike dispatch, without considering long-distance dispatch across time windows. The model allowed for a small portion of demands to be unmet, ignoring the potential revenue they could bring. The experimental results demonstrated the effectiveness of this optimization mechanism.

However, in actual business scenarios, operating companies can adopt various combination strategies to enhance customer loyalty and demand satisfaction rates. Operating companies encourage users with regular commuting needs to use their shared services through time-based membership cards. This strategy’s advantage makes demand more predictable but also has the disadvantage of reducing the average revenue per user. To meet the site’s demands as much as possible, operating companies will also deploy a certain surplus of bikes at sites to smooth out the dispatch volume. In future work, we will introduce net profit under multiple pricing strategies as an optimization goal. Operating costs also need to consider the maintenance and scrapping costs of bikes; thus, the safety stock at sites brings demand satisfaction revenue and also generates maintenance costs as a penalty item. In the actual dispatch process, we need to more clearly define site locations, rather than a geographical district, and the number of carrier vehicles is also subject to certain restrictions.

The method of this paper also has great application prospects in the optimization of public transportation systems. Public transportation systems, such as buses and subways, face similar challenges, including demand fluctuations, uneven resource allocation, and operating cost control. By effectively forecasting passenger demand at different times and locations, public transportation managers can better plan vehicle departure frequencies and route layouts to adapt to the spatiotemporal distribution of passenger demand. Public transportation dispatching must effectively allocate transportation capacity under limited resources to maximize demand satisfaction rates and operating efficiency. By deploying transportation capacity at key sites and times, it can better meet passenger demand and improve the overall efficiency of the system. Introducing comprehensive cost considerations in public transportation optimization, including vehicle maintenance, energy consumption, and labor costs, will help achieve a more economical and sustainable operating model.

## 6. Conclusions

This study aimed to address the supply–demand imbalance problem faced by the bike-sharing system in operation. For this purpose, we utilized spatiotemporal characteristics combined with T-GCN for shared bike demand prediction and designed a GA-based dispatch optimization scheme for shared bikes on this basis. The model first comprehensively considered users’ spatiotemporal demand and the dynamic changes in shared bike distribution through the Temporal Graph Convolutional Network, thereby effectively improving the accuracy of predictions. Secondly, we also fully considered characteristics such as the capacity of dispatch vehicles, distance restrictions, and the repeatability of dispatch routes to ensure that the dispatch scheme is more practically feasible and in line with actual conditions. In the experimental section, shared bike order data from the Beijing area were used to verify the outstanding performance of the T-GCN in short-term traffic prediction for shared bikes. Based on the prediction results, the model provided an optimized dispatch scheme, including the number of dispatch vehicles and the total dispatch cost. The research results show that the model and algorithm proposed in this paper effectively solved the problem of the periodic supply–demand imbalance in the bike-sharing system, improved system operation efficiency, reduced the vehicle idle rates and lowered the enterprise operating costs. This provides a solid foundation for future research on shared bike dispatch and offers practical solutions for the sustainable development of the shared economy field. 

## Figures and Tables

**Table 1:** Notations descriptions.

Notation	Description
*Z*	Total dispatch cost
$\left(x_{i},y_{i}\right)$	Node *i* coordinates
$Out_{i}$	The number of shared-bikes departing from node *i* at the start of dispatching
$In_{i}$	The number of shared-bikes entering node *i* at the end of dispatch.
$G_{i}$	The number of bikes required at node *i*
*D*	The maximum distance the dispatch vehicle can travel
$V_{m}$	The maximum loading capacity of the dispatch car *m*
$d_{ij}$	The distance between node *i* and node *j*
*C*	The unit distance cost of dispatching vehicles
$r_{mi}$	The number of bikes collected by vehicle *m* at node *i*
$p_{mi}$	The number of bikes placed by vehicle *m* at node *i*
$x_{mij}$	The decision variable. A binary variable with a value of 1 if vehicle *m* reaches node *j* from node *i*, and 0 otherwise

**Table 2:** Experimental data field descriptions.

Label	Meaning
orderid	Order number
orderid	User number
bikeid	Cycle number
biketype	Bike type
starttime	Order start time
geohashed_start_loc	Order start location
geohashed_end_loc	Order end location

**Table 3:** The validation loss associated with different GRU hidden sizes.

Hidden Sizes	Loss (Single-Step)	Loss (Two-Step)	Loss (Four-Step)
8	3.7664	5.0739	8.5486
16	2.5840	3.6724	4.7292
32	2.0192	2.9346	4.1562
64	1.3971	1.7332	3.4376
128	1.7933	2.0697	2.7761
256	1.9800	2.5230	2.8487

**Table 4:** Model parameters.

Parameters	Meaning	Value(s)
*D*	The maximum distance the dispatch vehicle can travel	10 (KM)
$V_{m}$	The maximum loading capacity of the dispatching car *m*	20, 15, 10 (/CAR)
*C*	The unit distance cost of dispatching vehicles	3 (CNY/KM)

**Table 5:** Genetic algorithm parameters.

Parameters	Meaning	Value(s)
*l*	Chromosome Encoding Length	2500
*p*	Population Size	100
$p_{c}$	Crossover Probability	0.95
$p_{m}$	Mutation Probability	0.05
iter	Maximum Iteration Count	200

**Table 6:** The T-GCN forecast results.

Metrics	30-min	60-min	120-min
MAE	0.4786	0.5342	0.8147
RMSE	2.2175	2.4909	3.7291
$R^{2}$	0.8974	0.8741	0.7131

**Table 7:** Genetic algorithm convergence iteration statistics from 7:00 AM to 10:00 PM.

Time Interval	Number of Iterations at Algorithm Convergence		
	Minimum	Maximum	Average
half hour	29	56	34
one hour	22	83	51

## Data Availability

The original data presented in the study are openly available in BienData at https://www.biendata.xyz/competition/mobike/data/.
